# Editorial: The Role of Genetic and Lifestyle Factors in Metabolic Diseases

**DOI:** 10.3389/fendo.2019.00475

**Published:** 2019-07-17

**Authors:** Shafqat Ahmad, Tarunveer S. Ahluwalia

**Affiliations:** ^1^Molecular Epidemiology Unit, Department of Medical Sciences, Uppsala University, Uppsala, Sweden; ^2^Preventive Medicine Division, Harvard Medical School, Brigham and Women's Hospital, Boston, MA, United States; ^3^Department of Nutrition, Harvard T.H. Chan School of Public Health, Boston, MA, United States; ^4^Steno Diabetes Center Copenhagen, Gentofte, Denmark

**Keywords:** gene environment interaction, obesity, type 2 diabetes, cardiovascular disease, genetic predisposition and association

The study of gene × environment interactions (GEI) in metabolic disease has become an active research area in the last few years (1). It has widely been accepted that both genetic and lifestyle risk factors associated with metabolic disease etiology (1) and the complex interplay of these factors influence metabolic disease risk (2, 3). Studies of GEI are very useful in gaining a better understanding of the biological pathways and the dose-response relationship, by obtaining better population-attributable risks, identifying individuals who may be more susceptible to metabolic disease risk, finding heterogeneity across studies and identifying novel disease susceptibility genes (4). Over the last 15 years, the field of genetic epidemiology has evolved from candidate gene, candidate GEI to genome-wide association studies (GWAS) and genome-wide interaction studies (GWIS) (1, 2). However, only a few reported GEI findings have been replicated which implies that the original findings might be false positives or that interaction estimates were cohort specific, or maybe the subsequent follow-up studies were underpowered and might have led to false positive findings (5).

In a systematic review, Haslam et al. summarized published literature about gene × sugar-sweetened beverage interactions in metabolic traits. Sugar-sweetened beverages include fruit drinks, soft drinks and other high energy soda drinks that are associated with an increased risk of metabolic disease. Authors reported several studies which support gene × sugar-sweetened beverage interactions and suggest that some individuals who consume higher sugar-sweetened beverages may be more susceptible to a greater risk of metabolic diseases. However, we should be careful in interpreting these findings as the interaction effect estimates are generally too small to have any clinically meaningful impact. GEI interaction evidence may also be helpful as a public health tool to encourage compliance with lifestyle modifications for high genetic risk individuals. Authors also reported that most published studies on gene × sugar-sweetened beverage interactions in metabolic traits have been conducted in a small sample and lack replication. It is worth noting that in observational studies the precision through which lifestyle exposure and outcome are assessed is usually very low and the observed studied genetic variants are also merely a proxy for the functional genetic locus, so, the underlying GEI are likely to be underestimated. Future studies should be conducted using samples based on prospective study designs and more diverse populations. Habberstad et al. examined the association between dietary intake and the sweet taste receptor (*TAS1R2*) associated genetic variant rs12033832. Authors observed modest associations between dietary intake and the *TAS1R2* rs12033832 genotype. Among study participants with a BMI ≥25 kg/m^2^, T-allele carriers of the *TAS1R2* rs12033832 genetic variant had a higher carbohydrate intake (*P*-value = 0.01) and a lower intake of fat (*P*-value = 0.03), however, authors were unable to observe these associations among individuals with a BMI <25 kg/m^2^. Ahmad et al. provides a comprehensive review on the published studies regarding GEI in metabolic traits among South Asians. The authors identified only seven published studies that have focused on GEI in obesity, type 2 diabetes, and cardiovascular related traits among South Asians. Some of the studies reported differences in metabolic response to caloric intake, smoking and physical activity that might be modified by genetic predisposition conferred to these metabolic traits. However, authors observed that most of the published GEI studies in South Asians were conducted in a relatively small sample and findings lack replication.

Raut and Khullar have highlighted the current knowledge on functional genomic long non-coding RNAs (lncRNAs) elements that were previously considered as transcriptional noise. However, in the past decade technology and next generation sequencing methods have provided strong evidence that lncRNAs have regulatory potentials that affect the genome and epigenome, impacting different diseases, an example of Gene × Gene Interactions and GEI. Authors have explained lncRNA types based on their genesis from the genome, and their mechanism of action in the pathogenesis of diabetes complications. Future research should focus more on understanding the mechanisms of newly identified lncRNAs, paving a way toward better therapeutics and the early diagnosis of diabetes complications. Zaharan et al. examined whether obesity related non-synonymous SNPs (in Europeans) are associated with physical activity (PA), adiposity related measures, and their interactions (SNP × PA) on adiposity outcomes in an ethnically diverse adolescent Malay population. In the gene adiposity association, only two SNPs *ADRB3* rs4994 and *MC3R* rs3827103 were associated with body fat percentage. The authors also reported a genetic risk score (GRS) × PA in adiposity measures. Authors emphasized the intervention opportunities among children with a greater genetic risk for obesity, while their PA levels are still modifiable. There is an emphasis on using polygenic risk score models (rather than single SNPs) for exercise interventions, which might be clinically beneficial. Krupková et al. demonstrate that gene-treatment interaction may predispose individuals differently toward metabolic alterations. Authors identified the *Zbtb16* (zinc finger and BTB domain containing 16) gene using spontaneously hypertensive congenic mice strains, that are known to affect blood pressure and metabolic alterations including insulin resistance. Authors provided evidence that a pharmaco-genetic intervention; where glucocorticoid-inducible transcription factor Zbtb16, if carrying a variant (among congenic mice models) shows metabolic alterations (increased plasma triglycerides and reduced insulin sensitivity). The *Zbtb16* variations can mediate DEX induced insulin resistance of muscle tissue. Thus, Krupková et al. demonstrate the importance of gene-drug interactions in metabolic outcomes, through congenic mice models, in addition to the identification of a new genes involved in the insulin resistance of muscle tissues.

In general, most GEI studies have been conducted using cross-sectional cohorts with small epidemiological samples, however, considering several large prospective study cohorts with available lifestyle and genetic information, including incident disease information, might make them improved resources for testing GEI in the future. However, GEI studies are prone to bias, chance, scale dependency and reverse causality which has been discussed elsewhere (6). Future GEI studies in metabolic traits should be expanded toward larger epidemiological settings with follow up data. It has already started in the form of consortia (group of multiple studies working together) which are helpful in harnessing the statistical power for assessing these associations. Large scale biobanking efforts, including deep phenotyping, are also initiating efforts such as the UK Biobank (https://www.ukbiobank.ac.uk/) and the Chinese Kadoorie Biobank (http://www.ckbiobank.org/site/), with data on around half a million individuals in each. In addition to this, large scale genetic and phenotypic information is also publicly available through the dbGAP or the database of genotypes and phenotypes (National Institute of Health (NIH) based effort; https://www.ncbi.nlm.nih.gov/gap/). We have demonstrated in the past that using publicly available (or consortium) data on metabolic traits not only strengthens the statistical power but may also acts as a validation step in a GWAS (7, 8) or a GWIS (9) setting. Another aspect that helps improve the results qualitatively is study power (sample size) calculations. Power calculations have now become an essential pre-requisite not only for the GEI studies but also for large scale genetic studies testing the association between low frequency or rare variants with an outcome of interest, as demonstrated by our recent study related to diabetic kidney disease genetics (10). Some of the reliable power calculation tools for GEI and genetic association studies are POWER (https://dceg.cancer.gov/tools/design/power) and GAS (http://csg.sph.umich.edu/abecasis/cats/gas_power_calculator/). In addition to the data resources and power calculations, we have previously demonstrated the optimal conditions to identify GEI in a multi-cohort setting (11). These include, (a) for a given interaction estimate, lifestyle exposure should be expressed on a continuous scale and if the exposure variable is dichotomized the categories should be equally prevalent, (b) variance in GRS should be large, (c) lifestyle exposure and GRS should be correlated, and (d) population outcome variance should be small (11).

In conclusion, the current Research Topic highlights some recent findings based on genetic and/or environmental factors and their impact on cardiometabolic traits in human or animal studies. It also discusses the strengths and limitations of such GEI studies, recommending how these studies can be improved, utilizing not only modern tools but also through the judicious use of publicly available resources, deep phenotyping, and longitudinal study designs. The translational value of GEI studies may also be enhanced by conducting studies across ancestries, diverse age groups and with additional complementing layers of data (including proteomics, metabolomics, and/or microbiome) in order to better understand disease pathogenesis. Altogether, these highlight the prospects of identifying high risk individuals from a population for the early management of metabolic disease risk and stepping forward toward personalized prevention and treatment strategies in the clinic.

## Author Contributions

All authors listed have made a substantial, direct and intellectual contribution to the work, and approved it for publication.

### Conflict of Interest Statement

The authors declare that the research was conducted in the absence of any commercial or financial relationships that could be construed as a potential conflict of interest.
